# HDA-YOLO: a hierarchical and densely-fused attention network for rice pest detection in complex agricultural environments

**DOI:** 10.3389/fpls.2026.1763650

**Published:** 2026-03-03

**Authors:** Shuo Yuan, Ying Duan, Hongting Su, Xinhui Zhou, Yinfeng Hao

**Affiliations:** 1 School of Computer and Information Engineering, Henan University, Kaifeng, Henan, China; 2 Henan Key Laboratory of Big Data Analysis and Processing, Henan University, Kaifeng, Henan, China

**Keywords:** attention mechanism, deep learning, mobile application, rice pest detection, smart rice agriculture

## Abstract

Rapid and intelligent identification of rice pests serves as the core sensing technology for precision plant protection and smart rice farming systems, providing critical support for intelligent cultivation decisions. To address the challenges of insufficient robustness and low precision of existing lightweight detection models in complex agricultural environments, this study proposes HDA-YOLO, an improved lightweight YOLOv8 model based on a hierarchical and densely-fused attention mechanism, for fast and high-precision pest detection. To enhance feature fidelity, the model incorporates asymmetric dynamic downsampling (ADDS) and a multi-scale cascade pre-fusion (MCPF) module into the backbone network. To achieve dynamic, content-aware feature fusion, a hierarchical attention-driven dense fusion network (HADF-Net) is constructed, integrating an intra-scale self-attention module (ISAM) and an inter-scale cross-attention module (ICAM). Furthermore, the C2f module is upgraded to a multi-scale context (MSC) module to improve adaptability to variations in target scale. Experimental results on the self-built RicePest_12 dataset demonstrate that HDA-YOLO, while maintaining a lightweight architecture (3.93M parameters, 12.02 GFLOPs), achieves significant improvements over the baseline YOLOv8n model, with mAP@50, F1-score, and Recall increasing by 2.4%, 3.8%, and 4.8%, respectively. In comparison with the Transformer-based RT-DETR-R18 model, HDA-YOLO achieves a 4.8 percentage points higher mAP@50, while its computational cost is only 22% and its parameter count is only 20% of RT-DETR-R18. Moreover, the proposed model has been successfully deployed on a mobile application, achieving real-time and accurate identification of field pests and demonstrating significant potential in the field of smart rice agriculture.

## 1 Introduction

Rice is one of the most vital food crops globally, and its stable production is intrinsically linked to food security and the livelihoods of farmers (Britto and Kronzucker, 2004). However, rice yield is severely threatened by diseases and infestations caused by a diverse range of insect pests, leading to substantial economic losses annually (Ali et al., 2021). Therefore, achieving efficient and accurate identification and monitoring of rice pests is not only a critical component of pest management and control but also holds profound significance for the advancement of smart agriculture (Santiteerakul et al., 2020; Savary et al., 2012).

Early studies primarily employed traditional machine learning algorithms, using handcrafted features such as scale-invariant feature transform (SIFT), histogram of oriented gradients (HOG), and local binary patterns (LBP) combined with classifiers such as support vector machine (SVM) and K-nearest neighbors (KNN) to achieve pest recognition (Waqas et al., 2025). Although these methods achieved certain accuracy under controlled conditions, their robustness in complex field environments was limited, being easily affected by variations in illumination, background interference, and the morphological diversity of pests (Liu et al., 2025). For instance, Xiong et al. (2024) pointed out that conventional pest detection methods are not only time-consuming and labor-intensive but also often fail to achieve real-time monitoring and rapid response, further highlighting their limitations in modern agricultural applications. Moreover, Zheng et al. (2024) emphasized that due to the high similarity among different pests, significant intra-class variations, and complex backgrounds, traditional methods struggle to accurately and quickly identify multiple rice pests, resulting in recognition accuracy significantly lower than that of deep learning models.

With the advancement of deep learning, convolutional neural networks (CNNs) and Vision Transformers have shown significant advantages in image recognition, leading to the widespread adoption of object detection algorithms in agricultural scenarios. Among these, one-stage object detectors, exemplified by the You Only Look Once (YOLO) series (Redmon et al., 2016; Redmon and Farhadi, 2018), have become a mainstream approach for agricultural pest identification due to their excellent balance between speed and accuracy (Badgujar et al., 2024). These models extract hierarchical features through deep backbone networks and leverage feature pyramid networks (FPN) (Lin et al., 2017) and their enhanced variants, like PANet (Liu et al., 2018), for multi-scale feature fusion, thereby enabling rapid localization and accurate classification of conventional targets.

To further enhance performance, a previous study has developed the MTD-YOLO model based on YOLOv8, which incorporates MobileNetV3 as the backbone network and integrates Triplet Attention and Dynamic Head, effectively improving feature representation capabilities and significantly increasing detection confidence and accuracy across multiple rice pest datasets (Zhang et al., 2025). Meanwhile, Rahman et al. (2020) proposed a CNN-based method for rice disease and pest recognition, demonstrating the feasibility of lightweight models for mobile deployment under complex and heterogeneous field conditions; Pan et al. (2023) further introduced the two-stage RiceNet method, which effectively enhances recognition robustness in challenging field backgrounds.

Nevertheless, when directly applying existing lightweight models, such as YOLOv8n, to real-world rice pest detection in the field, their performance is still constrained by a series of inherent challenges. First is the issue of information fidelity: pest images captured in the field often contain numerous small targets. As these weak visual features pass through the successive downsampling layers of a CNN, the sharp decline in spatial resolution can easily cause them to be submerged in background information, leading to irreversible information loss (Feng et al., 2023). Second is the challenge of feature discriminability: the background of rice paddies is exceedingly complex, and the color and texture of pests often bear a high resemblance to rice stems and leaves, creating a natural camouflage. This places stringent demands on the model’s ability to extract highly discriminative features from a cluttered environment (Hu et al., 2023). Finally, there is the trade-off between efficiency and accuracy: to enable real-time deployment on mobile or edge devices, the model must remain lightweight. However, this is typically achieved at the cost of network depth and width, which further exacerbates the aforementioned challenges (Hafiz, 2023).

To address these challenges, researchers have explored various avenues, such as designing more efficient feature fusion necks [e.g., BiFPN (Tan et al., 2020)] to enhance the interaction of multi-scale information, or embedding different types of attention mechanisms (Woo et al., 2018; Hu et al., 2020) into the network to guide the model’s focus toward critical feature regions. However, these improvements are often modular or plug-in in nature. Although such modular strategies can effectively optimize specific nodes, they remain insufficient to fundamentally overcome the systemic degradation and bottlenecks that feature information encounters throughout the end-to-end processes of extraction, transmission, and fusion. This underscores the need for a comprehensive and lightweight framework capable of systematically and synergistically addressing the aforementioned challenges.

The objective of this study is to address the challenges of rice pest detection in complex field environments by developing a lightweight yet high-performance object detection model, termed HDA-YOLO (Hierarchical and Densely-fused Attention YOLO). The core design philosophy of this model is the systematic and synergistic optimization of the three critical stages of the network—feature extraction, feature fusion, and feature interpretation—to maximize the fidelity and interaction efficiency of the information flow throughout the entire network. Specifically, four synergistic core innovations are introduced:

1. an Asymmetric Dynamic Downsampling (ADDS) module, which reduces information loss during the downsampling process in a content-adaptive manner;
2. a Multi-scale Cascade Pre-fusion (MCPF) module, employed at the end of the backbone to pre-fuse features for an enhanced output;
3. a Hierarchical Attention-Driven Dense Fusion Network (HADF-Net), which is built upon a dense topology and guided by a hierarchical attention mechanism, elevating feature fusion from a “static merge” to a “dynamic inference” process;
4. a Multi-Scale Context (MSC) module, which performs fine-grained analysis through parallel branches with multiple receptive fields to improve adaptability to varying target scales.

Through the deep coupling of these four innovations, HDA-YOLO constructs an end-to-end collaborative feature evolution architecture, enabling global information flow optimization from low-level perception to high-level semantic interpretation. On the self-built dataset, HDA-YOLO demonstrates significantly superior detection accuracy compared to the baseline model and has been successfully deployed in a WeChat Mini Program, demonstrating its significant application potential for intelligent agricultural monitoring scenarios.

## 2 Materials and methods

### 2.1 Data acquisition

Given the scarcity and inherent limitations of currently available public rice pest datasets, this study aimed to construct a more challenging dataset that closely reflects real-world agricultural scenarios. To achieve this, images from two large public datasets were integrated and curated: IP102 (Wu et al., 2019) and Pest_V2 (Quach et al., 2024). A dual-construction strategy was employed to enhance the dataset’s specificity and complexity.

First, acknowledging the significant morphological differences of rice pests across various life cycle stages, we subdivided the images within the Pest_V2 dataset. Different growth stages of the same pest (e.g., larva, adult) were annotated as distinct and independent categories. Second, to further increase the challenge posed by background complexity, pest images from the IP102 dataset that were both relevant to rice and featured more intricate backgrounds were manually selected and extracted to supplement the new dataset.

**Figure 1:**
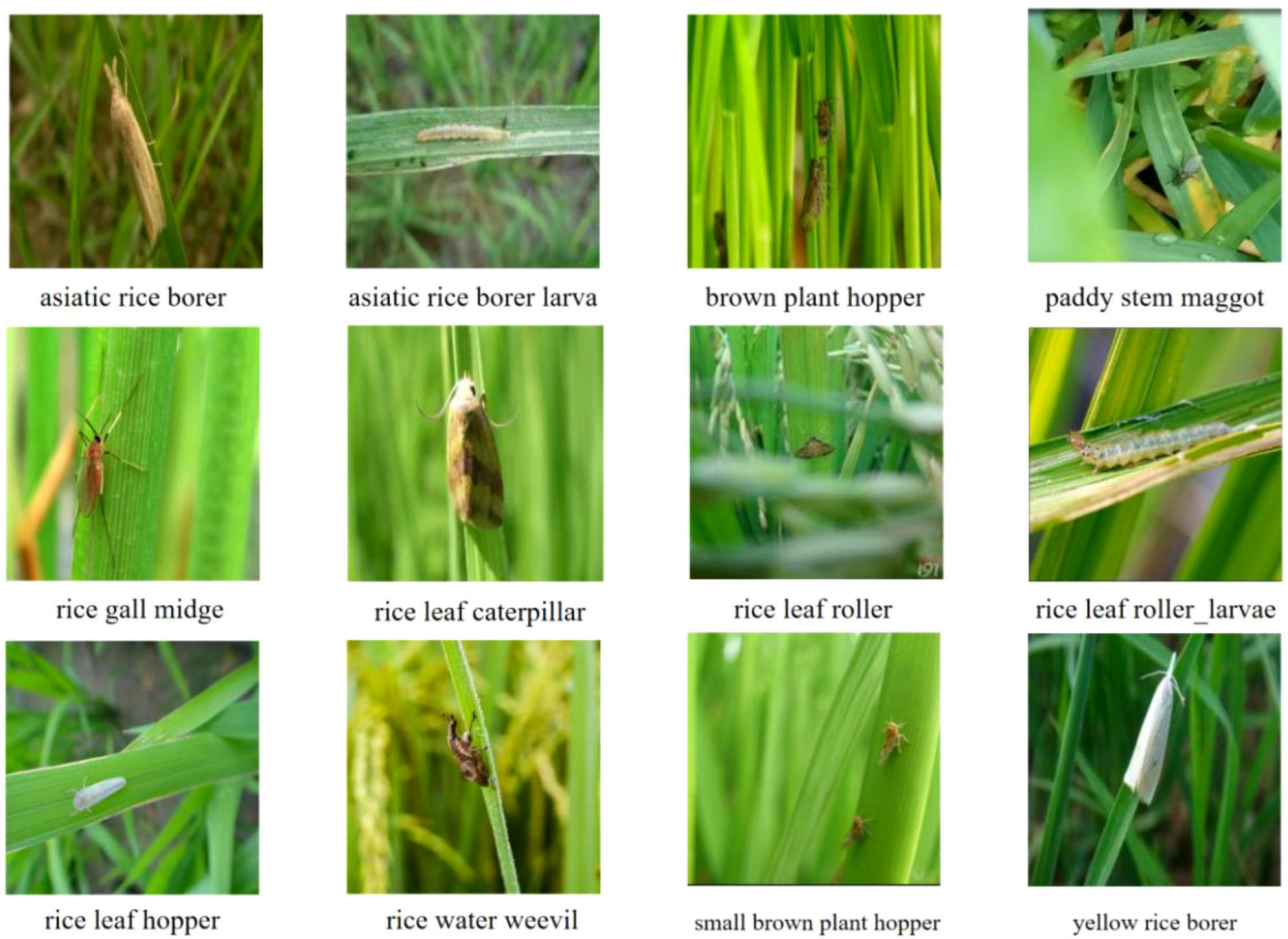
Examples of the 12 rice pest species used in this study.

Through the aforementioned process, a customized dataset named RicePest_12 was ultimately constructed. This dataset comprises a total of 2,807 high-quality images, covering 12 common categories of rice pests, such as the Asiatic Rice Borer, Brown Plant Hopper, and Paddy Stem Maggot (as shown in **Figure 1**). The creation of this dataset provides a high-quality and challenging experimental foundation for our research, ensuring the reliability and validity of the model evaluation.

### 2.2 Data preprocessing

Although the constructed RicePest_12 dataset is of high quality and presents significant challenges, its total volume of 2,807 images is still relatively insufficient. Furthermore, the dataset exhibits a notable disparity in the number of samples among different categories, presenting a typical long-tail distribution. This data imbalance issue can cause the model to develop a bias towards the majority classes during training, thereby weakening its generalization ability for rare categories and potentially leading to overfitting.

To address the aforementioned issues, this study employed a comprehensive data augmentation strategy to expand the dataset and balance the class distribution. A variety of data augmentation techniques were applied to the original images, including geometric transformations (e.g., random horizontal and vertical flips, rotation, scaling, translation, and perspective transformation) and appearance transformations (e.g., blurring). Furthermore, to enhance the model’s ability to discern complex backgrounds and reduce the false detection rate, unlabeled pure background images were strategically introduced into the training set as negative samples.

Through this augmentation and expansion, the final dataset size was increased from 2,807 to 6,374 images. The distribution of sample quantities across the various categories is detailed in **Table 1**. Prior to model training, this augmented dataset was randomly partitioned into training, validation, and test sets according to an 8:1:1 ratio.

**Table 1:** Distribution of images across categories in the RicePest_12 dataset before and after data augmentation.

Category	Original number of images	Augmented number of images
Asiatic Rice Borer (adult)	187	500
Asiatic Rice Borer (larvae)	237	500
Brown Plant Hopper	251	500
Paddy Stem Maggot	67	500
Rice Gall Midge	124	500
Rice Leaf Caterpillar	103	500
Rice Leaf Roller (adult)	172	500
Rice Leaf Roller (larvae)	574	574
Rice Leaf Hopper	237	500
Rice Water Weevil	420	500
Small Brown Plant Hopper	245	500
Yellow Rice Borer	190	500
Background	0	300

### 2.3 Model overview

**Figure 2:**
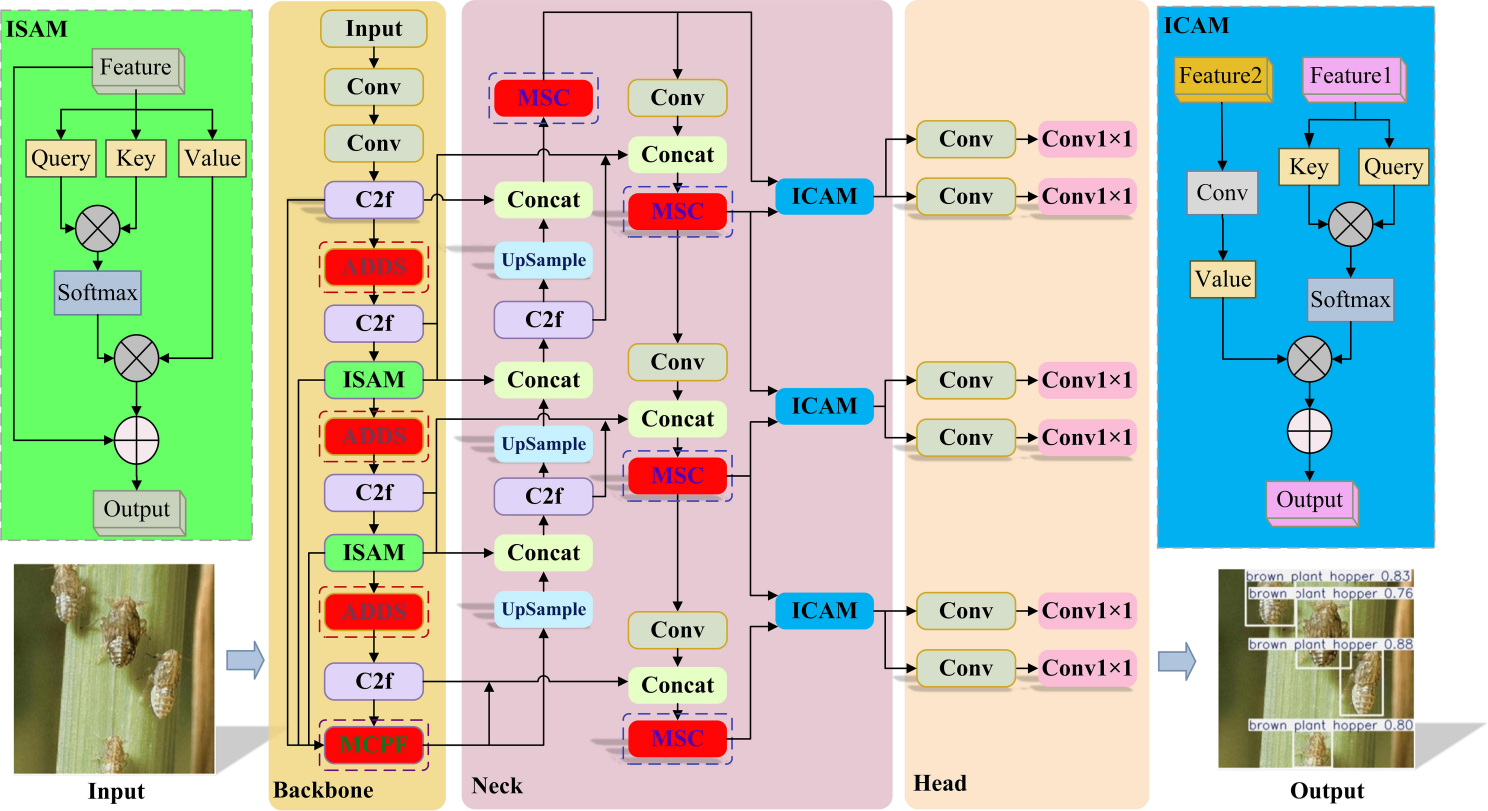
Architecture of HDA-YOLO, featuring a backbone with Asymmetric Dynamic Downsampling (ADDS) and Multi-scale Cascade Pre-fusion (MCPF) modules for enhanced feature fidelity, and a neck with a Hierarchical Attention-Driven Dense Fusion network (HADF-Net) and Multi-Scale Context (MSC) module supporting dynamic multi-scale fusion.

This paper proposes an object detection framework named HDA-YOLO, as illustrated in **Figure 2**. This framework is designed to enhance the detection accuracy of multi-scale rice pests against complex backgrounds, achieving an effective integration of high precision and a lightweight structure. The overall architecture of the model is based on YOLOv8, but its core lies in the systemic, end-to-end optimization of the network’s information flow.

In the backbone network, HDA-YOLO leverages the synergistic action of an ADDS module and a MCPF module. This combination enhances the fidelity of feature extraction right from the source, providing a high-quality feature foundation for subsequent network layers. Building upon this, a novel feature fusion system was constructed: the HADF-Net. Structurally, this network employs a dense aggregation topology, utilizing cross-layer “shortcuts” to counteract information dilution. Mechanistically, it is guided by hierarchical attention, which uses an intra-scale self-attention module (ISAM) to enhance features and an inter-scale cross-attention module (ICAM) to achieve an intelligent mapping from fused features to precise predictions. Simultaneously, to further bolster the network’s multi-scale analysis capabilities during feature fusion, the C2f module in the model’s neck has been upgraded to a MSC module. The MSC module performs fine-grained analysis through parallel branches with multiple receptive fields, significantly improving the model’s adaptability to variations in target scale.

Regarding the training strategy, to enhance the model’s generalization ability to real-world field environments, images of pests under diverse lighting conditions and at various growth stages, as well as pictures with a multitude of field backgrounds, were incorporated into the dataset. This was done to increase the overall diversity and complexity of the data.

### 2.4 Model improvements

#### 2.4.1 MCPF module

To address the issue of shallow spatial information loss in the traditional spatial pyramid pooling fusion (SPPF) (He et al., 2015) module, which results from it operating solely on the single, deepest feature map (P5), this study proposes the MCPF module, and its core idea is to re-architect the terminal stage of the backbone network from a simple “context aggregation” unit into an active “multi-source feature pre-fusion” unit. The objective is to generate a more comprehensive and detail-rich feature map before the inputs proceed to the detection neck by cascading the fusion of multi-scale features from P2 through P5.

**Figure 3:**
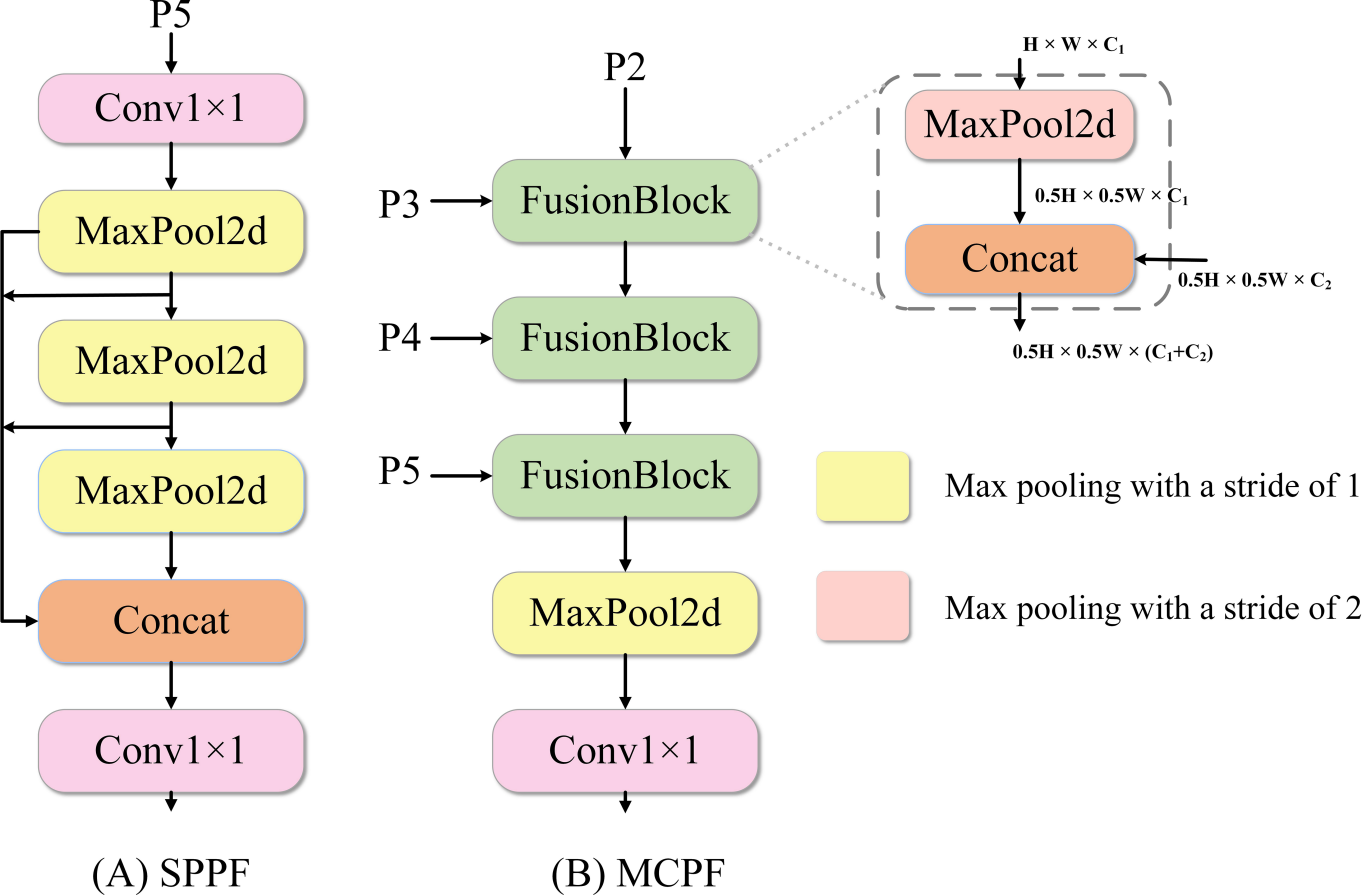
The architecture of the proposed MCPF module in comparison with the SPPF module. **(A)** SPPF operates on a single input feature map. **(B)** MCPF integrates multi-scale feature maps in a cascade.

As illustrated in **Figure 3**, MCPF abandons the single-input constraint, instead accepting feature maps from multiple stages of the backbone network (P2, P3, P4, P5) as parallel inputs. Internally, it follows a “downsample-and-fuse” cascade workflow, progressively integrating high-resolution features into the deeper feature stream. The computational process of the module is formulated as shown in Equations 1–4:

(1) $$\begin{array}{c} H_{3}={\text{M}}_{\text{s}=2}(\text{Concat}[M_{s=2}(P_{2}),{\text{P}}_{3}]) \end{array}$$

(2) $$\begin{array}{c} H_{4}={\text{M}}_{\text{s}=2}(\text{Concat}[H_{3},{\text{P}}_{4}]) \end{array}$$

(3) $$\begin{array}{c} H_{5}=\text{Concat}[H_{4},{\text{P}}_{5}] \end{array}$$

(4) $$\begin{array}{c} Y_{\text{MCPF}}={\text{Conv}}_{1\times 1}(M_{\text{s}=1}(H_{5})) \end{array}$$

where ${\text{P}}_{\text{i}}\in {\text{R}}^{\text{B}\times {\text{C}}_{\text{i}}\times {\text{H}}_{\text{i}}\times {\text{W}}_{\text{i}}}$, ${\text{M}}_{\text{s}=\text{k}}(.)$ represents the max-pooling operation with a stride of k, and the final output is ${\text{Y}}_{\text{MCPF}}$.

#### 2.4.2 ADDS module

**Figure 4:**
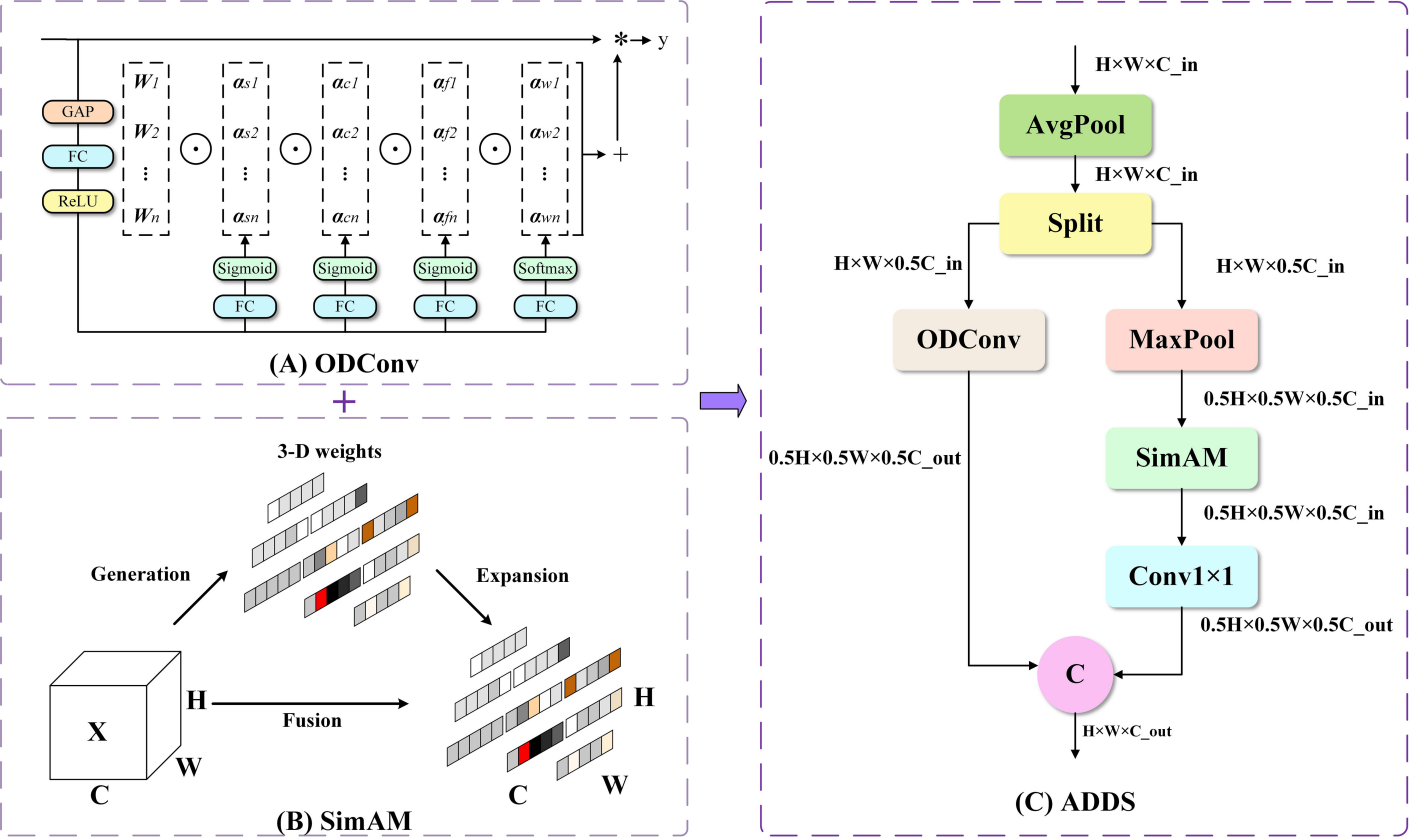
The architecture of the proposed ADDS module and its key components. **(A)** The ODConv block. **(B)** The SimAM attention mechanism. **(C)** The overall architecture of the ADDS module.

Starting from the fundamental operations of the network, this study designs an ADDS module. As illustrated in **Figure 4**, this module begins by applying average pooling to the input feature channels and then splits them into two halves. These halves are processed through two complementary, parallel paths:

One path employs Omnidimensional Dynamic Convolution (ODConv) (Li et al., 2022) to transform and downsample the features in a content-adaptive manner, aiming to preserve rich patterns and textures; The other path uses a combination of max-pooling and the SimAM attention mechanism (Yang et al., 2021) to focus on capturing and refining the most salient core structural features. Finally, the outputs of these two paths are concatenated, generating a downsampled feature map with higher information fidelity that contains both adaptively transformed information and preserved key salient structures. The overall computational process of this module can be expressed by the following Equations 5–8:

(5) $$\begin{array}{c} X=AvgPool(X_{in}) \end{array}$$

(6) $$\begin{array}{c} Y_{a}=F_{ODConv}(X_{a}) \end{array}$$

(7) $$\begin{array}{c} Y_{b}={\text{Conv}}_{1\times 1}(A_{\text{SimAM}}(\text{MaxPool}(X_{b}))) \end{array}$$

(8) $$\begin{array}{c} Y_{\text{ADDS}}=\text{Concat}(Y_{a}{\text{,Y}}_{b}) \end{array}$$

where $X_{a}$ and $X_{b}$ are the two parts of the feature X after it has undergone average pooling and been split. $Y_{a}$ and $Y_{b}$ represent the outputs from the dynamic convolution downsampling branch and the salient feature retention branch, respectively. The final output is denoted as $Y_{\text{ADDS}}$.

#### 2.4.3 HADF-Net

To address the limitations of networks like PANet and BiFPN, which are employed by YOLOv8 and are constrained by static fusion pathways and issues with information fidelity, the study proposed a novel HADF-Net. The core of this network lies in its ability to elevate the feature fusion process from a passive “static merge” into an active, content-aware “dynamic inference.” This is achieved by using a hierarchical attention mechanism as the central driving force, built upon a dense aggregation topology. Consequently, the network can better adapt to complex and highly variable detection scenarios.

**Figure 5:**
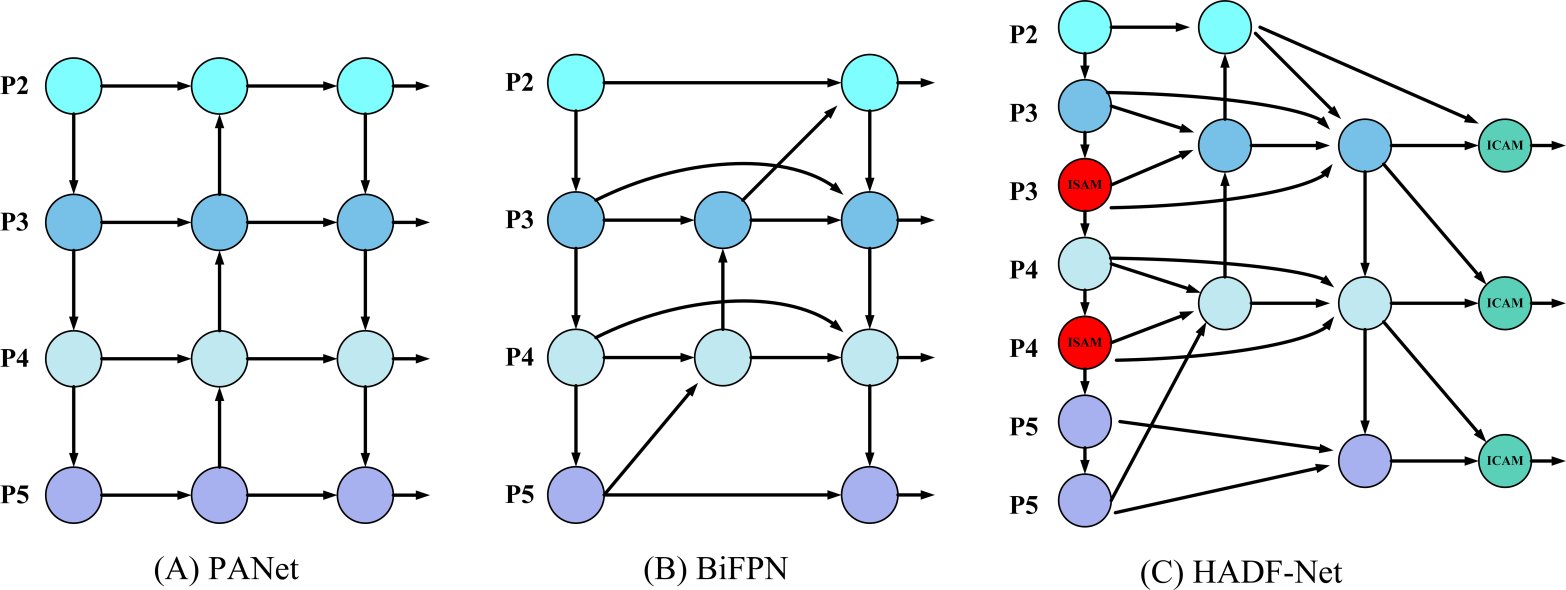
Comparison of feature fusion network architectures. **(A)** PANet. **(B)** BiFPN. **(C)** Our proposed HADF-Net.

As illustrated in **Figure 5**, HADF-Net structurally establishes denser, wider-span cross-layer “shortcuts” than those found in BiFPN. This design repeatedly re-injects the original high-resolution features from the backbone network into the bottom-up fusion path. Such a dense aggregation topology creates more direct pathways for information flow within the network. This physically ensures the maximum possible fidelity of shallow spatial details and provides an extremely information-rich feature pool for the subsequent attention mechanisms to operate on.

**Figure 6:**
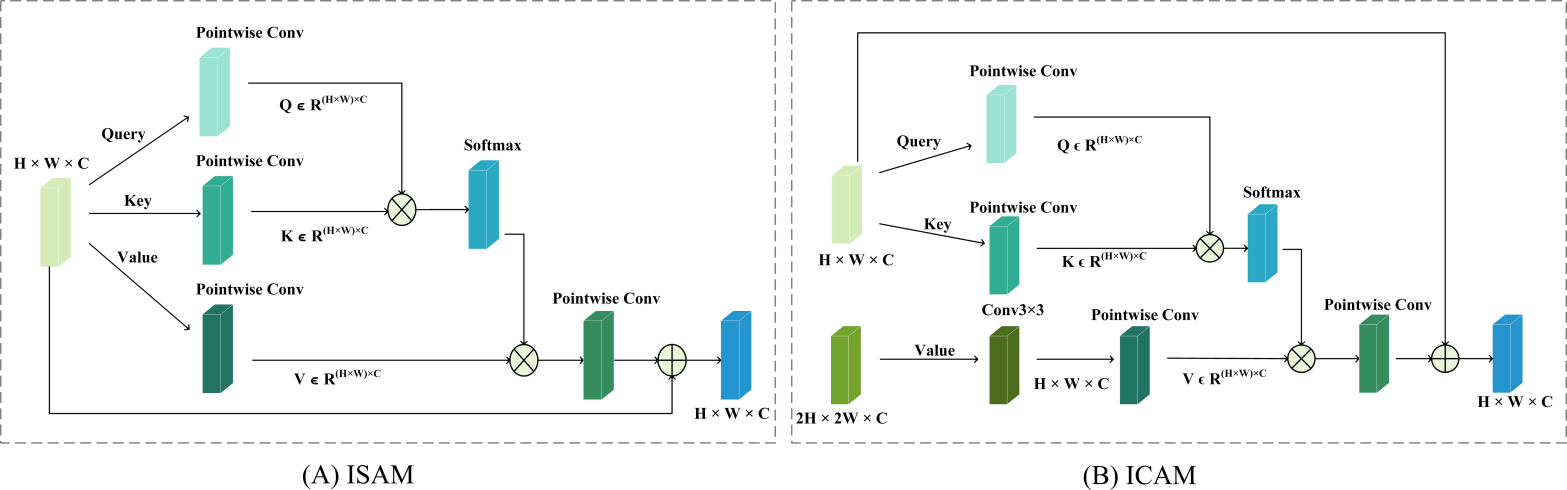
The core modules of the Hierarchical Attention mechanism. **(A)** ISAM module. **(B)** ICAM module.

Simultaneously, the HADF-Net’s information flow is governed by a hierarchical attention mechanism inspired by the Transformer(Vaswani et al., 2017). As shown in **Figure 6**, this mechanism achieves dynamic feature enhancement and fusion by deploying specific types of attention at different levels. First, before features enter the fusion path, an ISAM is used to perform global contextual enhancement on the features at each level. Its computation is as follows (Equation 9):

(9) $$\begin{array}{c} Y_{self}=X+softmax(\frac{(X^{T}W_{q}){\left(X^{T}W_{k}\right)}^{T}}{\sqrt{d_{k}}})(X^{T}W_{v}) \end{array}$$

Subsequently, at the fusion nodes within the neck of the network, ICAM replaces traditional static fusion methods. This module utilizes the deep features, ${\text{X}}_{\text{deep}}$, as a “query” to dynamically “probe” and integrate information from the aligned shallow features, ${\text{X}}_{\text{shallow}}^{'}$. The core computation are as follows (Equations 10, 11):

(10) $$\begin{array}{c} \text{X}{'}_{\text{shallow}}={\text{Conv}}_{\text{down}}(X_{\text{shallow}}) \end{array}$$

(11) $$\begin{array}{c} Y_{\text{cross}}=X_{deep}+softmax(\frac{(X_{deep}^{T}W_{q}){\left(X_{deep}^{T}W_{k}\right)}^{T}}{\sqrt{d_{k}}})({\left(X_{\text{shallow}}^{'}\right)}^{T}W_{v}) \end{array}$$

where $d_{k}$ is the dimension of the key vectors, used for scaling the dot-product results.

This hierarchical attention design, which operates on an “enhance-then-select” principle, enables the HADF-Net to intelligently adjust the information flow based on the input content. Consequently, it provides a higher-quality and more information-focused feature map for the final prediction.

#### 2.4.4 MSC module

**Figure 7:**
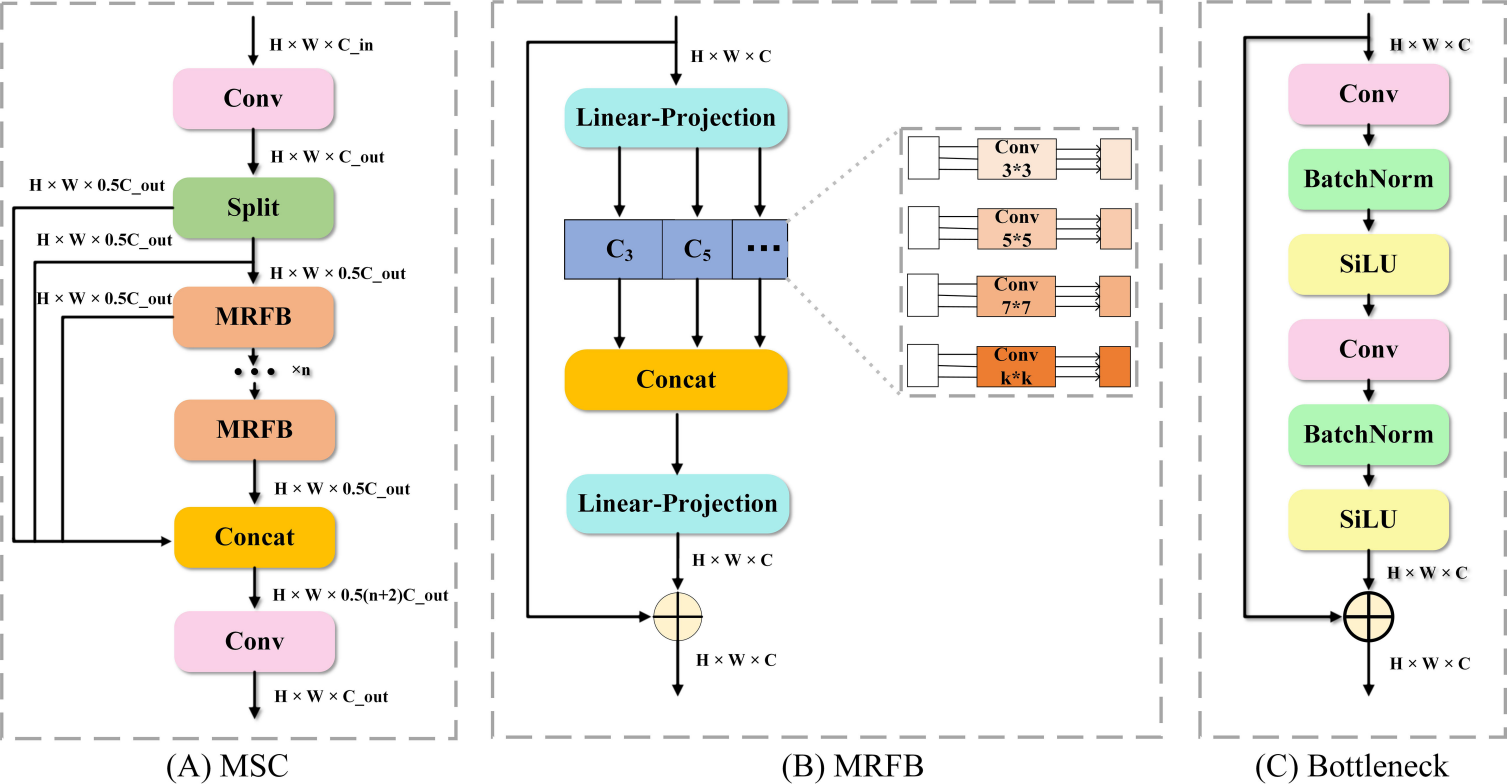
Architectural comparison of the proposed MSC module and the original Bottleneck. **(A)** The proposed MSC module. **(B)** The core MRFB. **(C)** The original Bottleneck unit.

As illustrated in **Figure 7**, to optimize the multi-scale contextual analysis capability during feature fusion, this study proposes a MSC module to replace the original C2f module. The primary innovation of the MSC lies in its core computational unit: the bottleneck unit from the C2f module is replaced by a multi-receptive field block (MRFB).

The Bottleneck in the C2f structure is formed by serially connected convolutions of a single size, and the homogeneity of its receptive field often makes it difficult to effectively handle variations in object scale. In contrast, the MRFB within the MSC employs a multi-branch parallel structure. It extracts multi-granularity features by using group convolution kernels of different sizes, thereby forming multiple subspaces with different receptive fields. This design allows the module to better adapt to changes in object scale.

Its subspace fusion strategy is inspired by the Inception architecture (Szegedy et al., 2015), employing channel concatenation to merge the subspaces from different receptive fields into a high-dimensional, nonlinear feature space. This enhances the multi-scale representation capability within a single feature map. Specifically, the module is designed with parallel 3×3, 5×5, and 7×7 group convolutions. Subsequently, these subspaces are fused via channel concatenation, followed by batch normalization and an activation function. Finally, a 1×1 convolution is used for information refinement. This core process can be mathematically represented as (Equation 12):

(12) $$\begin{array}{c} Y_{MRFB}=X+Conv_{1\times 1}(Concat[\left\{GroupConv_{k\times k}(Conv_{1\times 1}(X)\right){\}}_{k\in 3,5,7,\ldots }]) \end{array}$$

where $GroupConv_{k\times k}$(.) denotes the group convolution operation with a kernel size of k×k.

### 2.5 Model performance evaluation

#### 2.5.1 Experimental setup

The model training for this study was conducted using the PyTorch framework on GPU computing resources. The detailed configurations of the experimental environment and the model’s hyperparameters are presented in **Table 2**.

**Table 2:** Experimental environment and hyperparameter settings for model training.

Configuration	Parameter
Operating System	Ubuntu20.04
Deep Learning Framework	PyTorch 1.10.0
CUDA Version	11.3
Python Version	3.8
GPU	V100-32GB (32GB)
Epochs	100
Input Image Size	512 × 512
Batch Size	32
Dataloader Workers	4
Learning Rate	0.01
Optimizer	Adam

#### 2.5.2 Evaluation metrics

To comprehensively evaluate the performance of the model, five key metrics were adopted: Precision, Recall, mean Average Precision (mAP), F1-score, and Giga Floating-Point Operations (GFLOPs). These evaluation metrics are defined in Equations 13–17 as follows:

(13) $$\begin{array}{c} Precision=\frac{TP}{TP+FP} \end{array}$$

(14) $$\begin{array}{c} Recall=\frac{TP}{TP+FN} \end{array}$$

(15) $$\begin{array}{c} AP=\int_{0}^{1}P(R)dR \end{array}$$

(16) $$\begin{array}{c} mAP=\frac{1}{C}\sum_{i=1}^{C}AP_{i} \end{array}$$

(17) $$\begin{array}{c} F1-score=\frac{2\times Recall\times Precision}{Recall+Precision} \end{array}$$

where TP (True Positives) is the number of correctly predicted positive samples, FP is the number of incorrectly predicted positive samples, and FN is the number of incorrectly predicted negative samples. C represents the total number of detection classes, and AP is the Average Precision for a single class. Furthermore, GFLOPs is used to measure the computational complexity of a model, defined as the number of billion floating-point operations required to complete a single forward pass. A lower GFLOPs value indicates higher computational efficiency and a lower demand on hardware resources.

## 3 Results and analysis

### 3.1 Ablation experiments

To systematically validate the effectiveness of each innovative design proposed in this study, a series of detailed ablation experiments were conducted using YOLOv8n as the baseline model. For ease of presentation and analysis, the four modules—MCPF, ADDS, MSC, and HADF-Net—are abbreviated as A, B, C, and D, respectively. The ablation study was performed using the control variable method. All experiments were conducted on our self-built rice pest dataset, with completely consistent data augmentation strategies and experimental environments maintained throughout to ensure the fairness of the comparisons.

The ablation study results in **Table 3** clearly reveal how our proposed modules work synergistically to progressively enhance the model’s performance. In the initial stage, integrating module A (MCPF) enhanced the model’s ability to capture potential targets, increasing Recall by 0.7 percentage points, though this also introduced some discriminative ambiguity, causing a slight dip in mAP@50 to 87.2%. Building on this, the introduction of module B (ADDS) effectively resolved this issue. As a highly efficient downsampling module, ADDS significantly refined the features, greatly strengthening the model’s discriminative power. This led to a 1.2 percentage point leap in mAP@50 to 88.4%, with Precision also reaching its peak of 91.0%. Furthermore, the addition of module C (MSC) promoted a rebalancing between Precision and Recall. The Recall was observed to improve by 1.3 percentage points, while mAP@50 concurrently exhibited a steady increase, reaching 89.0%. Moreover, the more comprehensive metric, mAP@50-95, also demonstrated an improvement of 1.3 percentage points. Finally, with the integration of module D (HADF-Net), the complete HDA-YOLO model achieved optimal performance across all metrics. Precision returned to its peak of 91.0%, while Recall achieved a significant growth of 3.8 percentage points, pushing the F1-score to 88.2%. The mAP@50 also successfully reached 90.0%. This series of performance evolutions provides strong evidence that our four proposed modules each contribute unique and crucial performance gains, working in synergy to ultimately achieve a leap in the model’s overall performance.

**Table 3:** Ablation study of HDA-YOLO components.

Model	A	B	C	D	Precision	Recall	mAP@50	mAP@50-95	F1-score
Baseline(YOLOv8n)					0.882	0.808	0.876	0.618	0.844
Baseline+A	✓				0.872	0.815	0.872	0.616	0.843
Baseline+A+B	✓	✓			0.910	0.805	0.884	0.623	0.854
Baseline+A+B+C	✓	✓	✓		0.889	0.818	0.890	0.636	0.852
HDA-YOLO (Ours)	✓	✓	✓	✓	0.910	0.856	0.90	0.642	0.882

[A checkmark (√) indicates that the corresponding module was added to the configuration. A, B, C, and D represent the MCPF, ADDS, MSC, and HADF-Net modules, respectively.]

### 3.2 Comparative experiments with different models

To comprehensively evaluate the overall performance of the HDA-YOLO model, fair comparative experiments were conducted against several current mainstream lightweight object detection models on our self-built rice pest dataset. The comparison included models such as EfficientDet-D0, the Transformer-based RT-DETR-R18, and several representative models from the YOLO series. All models were evaluated using a unified training strategy and testing environment. The experimental results are summarized in **Table 4**.

**Table 4:** Performance comparison with state-of-the-art lightweight models.

Model	Precision	Recall	mAP@50	mAP@50-95	F1-score	GFLOPs	Parameters(M)
EfficientDet-D0	0.848	0.606	0.649	0.377	0.707	23.37	10.10
RT-DETR-R18	0.885	0.809	0.852	0.610	0.845	53.85	19.51
YOLOv5n	0.860	0.738	0.840	0.581	0.795	7.19	2.51
YOLOv8n	0.882	0.808	0.876	0.618	0.844	8.21	3.01
YOLOv9t	0.888	0.744	0.852	0.601	0.81	7.86	**2.01**
YOLOv11n	0.828	0.771	0.843	0.594	0.799	**6.45**	2.59
HDA-YOLO	**0.910**	**0.856**	**0.90**	**0.642**	**0.882**	12.02	3.93

(The best performance for each metric is highlighted in bold.)

As shown by the detection metrics in **Table 4**, the proposed HDA-YOLO model significantly outperforms all other compared models across all core metrics related to detection accuracy. Compared to the YOLOv8n baseline, our model achieves notable improvements of 2.4 and 3.8 percentage points in mAP@50 and F1-score, respectively. Furthermore, its Precision and Recall are higher by 2.8 and 4.8 percentage points. It is particularly noteworthy that when compared to the Transformer-based RT-DETR-R18, although the latter also exhibits strong performance, its computational cost (53.85 GFLOPs) and parameter count (19.51M) are far higher than our model’s. HDA-YOLO achieves superior detection accuracy while using only approximately 22% of the computational resources and 20% of the parameters, demonstrating the remarkable efficiency of deep optimization within a CNN architecture. Furthermore, when compared with YOLOv9t, which is extremely optimized for a low parameter count, HDA-YOLO leverages a moderate resource investment (3.93M vs. 2.01M parameters) in exchange for a substantial performance advantage of nearly 5 percentage points in mAP@50 (90.0% vs. 85.2%). Meanwhile, compared with YOLOv11n, which has the lowest computational complexity (6.45 GFLOPs), its Precision, Recall, and mAP@50 are 82.8%, 77.1%, and 84.3%, respectively. In contrast, HDA-YOLO improves these metrics to 91.0%, 85.6%, and 90.0% with only a slight increase in computational cost, demonstrating a more favorable trade-off between performance and efficiency.

The above experimental results demonstrate that for the task of rice pest detection, HDA-YOLO establishes a new benchmark for maximizing detection accuracy within an acceptable computational budget. By delivering significant precision gains (ranging from 2.4 to 6.0 percentage points in mAP@50) compared to various mainstream lightweight models—including the YOLO series and the Transformer-based RT-DETR-R18—the model demonstrates a superior performance-efficiency trade-off. Although there is a modest increase in GFLOPs and parameter count, HDA-YOLO’s overall complexity remains strictly within the lightweight range (12.02 GFLOPs and 3.93M parameters). This highlights its significant application potential for intelligent agricultural monitoring scenarios with high demands on detection accuracy.

**Figure 8:**
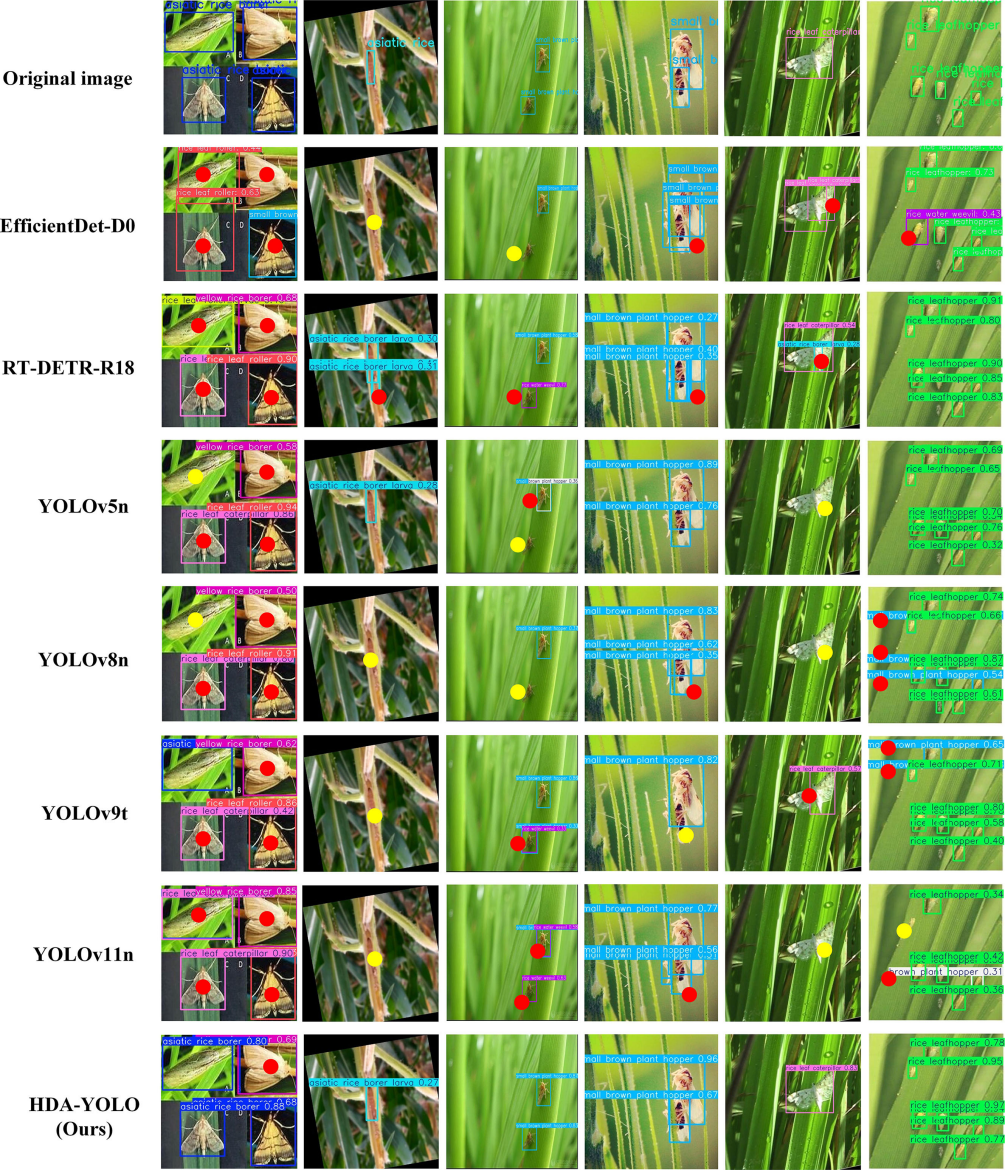
Qualitative comparison of detection results on challenging images. (Red solid circles indicate false detections, while yellow solid circles highlight missed detections.).

To visually demonstrate the practical advantages of HDA-YOLO, **Figure 8** provides a comparison of its detection results against those of several mainstream lightweight models on a set of challenging images. It can be clearly observed that HDA-YOLO, through its systematic end-to-end optimization, exhibits significant superiority across multiple complex scenarios. For the detection of small and dense objects (as shown in the fourth and sixth columns of **Figure 8**), HDA-YOLO accurately detects and distinguishes each individual, effectively resolving issues of missed detections, false detections, and inaccurate bounding box localization that are present in other models. This performance improvement reflects the synergistic effect of its dense fusion topology and the MSC module in combating information dilution and refining multi-scale analysis. Simultaneously, in distinguishing between the background and morphologically similar targets (as seen in the first, second, and third columns of **Figure 8**), HDA-YOLO demonstrates stronger robustness. It accurately identifies pests while effectively suppressing interference from similar-looking stems and leaves in the background. This enhanced capability stems from the effectiveness of the hierarchical attention mechanism and the ADDS module in optimizing feature representation. These qualitative results visually validate the synergistic effect of HDA-YOLO’s innovations in improving model accuracy while reducing false and missed detection rates, proving its significant application potential for pest monitoring in smart agriculture.

**Figure 9:**
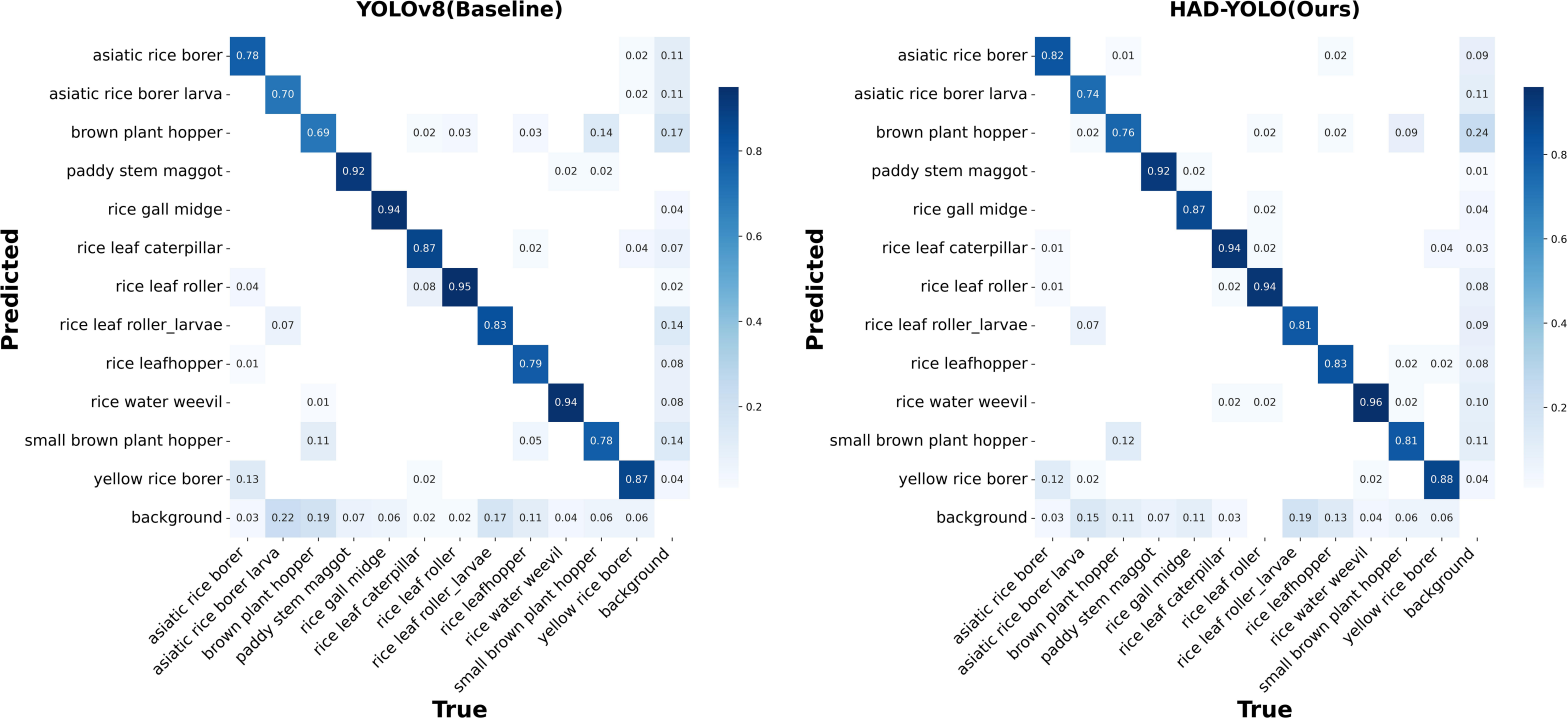
Comparison of confusion matrices: baseline model (left) vs. our model (right).

To evaluate model performance at a fine-grained level, **Figure 9** once again presents a comparison of the confusion matrices between the YOLOv8 baseline model (left) and the proposed HDA-YOLO model (right) on the test set. Confusion matrices not only provide an intuitive representation of overall classification accuracy but also reveal misclassification patterns and confusion relationships among different classes.

As can be seen from the statistical results in **Figure 9**, the overall detection accuracy of the proposed HDA-YOLO in this study is significantly better than that of the YOLOv8 baseline model. As can be seen from the comparison, the proposed model exhibits a significant increase in the proportion of main diagonal entries (indicating correct classifications) and a noticeable reduction in off-diagonal misclassifications. This systematically validates its superior recognition robustness and classification reliability in complex scenarios. Specifically, the YOLOv8 model achieved the lowest detection accuracy of only 69% for the brown plant hopper category, followed by the asiatic rice borer larva category with an accuracy of 70%. This outcome is likely attributable to the high morphological similarity between these two pest species and the rice field background, resulting in substantial pixel-level interference in the images. In contrast, the proposed model in this study achieved detection accuracies of 76% and 74% for the two aforementioned categories, respectively. These results demonstrate a notable improvement in accuracy and highlight the model’s enhanced capability to extract pest features in complex field environments. Nevertheless, despite the significant overall improvement in accuracy, a small number of failure modes can still be observed from the confusion matrix. One type of error arises from background confusion; for example, the brown plant hopper still exhibits an approximately 24% miss-detection rate due to its strong camouflage and high visual similarity to the background. Another source of error stems from inter-class similarity, where feature overlap among morphologically similar species leads to misclassification; for instance, about 12% of brown plant hoppers are misclassified as small brown plant hoppers, and 12% of asiatic rice borers are misidentified as yellow rice borers. These results indicate that there remains room for further optimization when addressing extreme camouflage conditions and fine-grained recognition of very small targets.

### 3.3 Heatmap analysis of the model’s internal states

To further enhance the interpretability of the improved model and provide intuitive qualitative evidence for our proposed innovations, this study focuses on the analysis of the MCPF module, the MSC module, and the HADF-Net. By employing the Grad-CAM technique (Selvaraju et al., 2019), we generate comparative feature heatmaps from before and after processing by our modules, or against the baseline modules, to improve the model’s explainability. Grad-CAM (Gradient-weighted Class Activation Mapping) is a technique designed to explain the decision-making process of convolutional neural networks. This method generates heatmaps to visualize the key image regions the model focuses on when making a decision, thereby helping to understand the reasoning behind a specific prediction.

**Figure 10:**
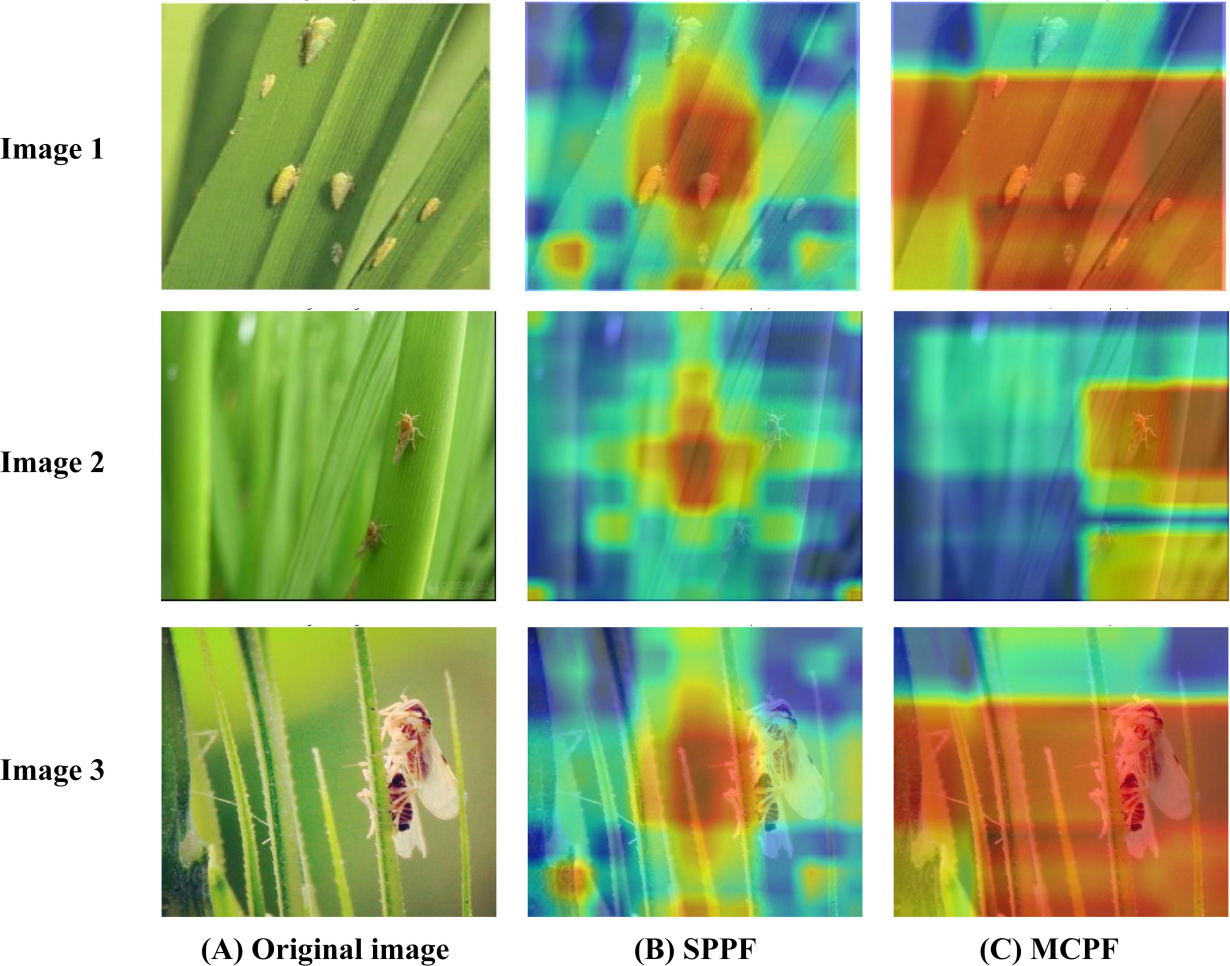
Class activation map (CAM) comparison of SPPF and MCPF. **(A)** Original image. **(B)** SPPF heatmap. **(C)** MCPF heatmap.

In the heatmaps generated by Grad-CAM, deep red indicates that the model pays extremely high attention to that image region, making it a key basis for its decision. Yellow areas represent regions of lower, yet still significant, importance, while blue areas indicate that the features in that location have a minimal impact on the model’s prediction. As shown in **Figure 10**, the feature heatmaps output at the terminal end of the backbone network by the baseline YOLOv8n’s SPPF module and the proposed MCPF module were first compared. The heatmap from the baseline’s SPPF module exhibits distinct global context characteristics and a strong center bias. Its activation area broadly covers the center of the image but lacks a precise response to specific, particularly small-sized, pest targets, for which the activation signal is weak. In contrast, the MCPF module proposed in this paper generates a feature map with extremely high spatial information fidelity by performing a cascade fusion of multi-scale features from P2 to P5. Its heatmap activation regions are more concentrated and effectively cover the key areas of the target. This result demonstrates that the design of the MCPF effectively overcomes the issue of spatial detail loss in deep networks, providing a higher-quality feature foundation with richer localization information for the subsequent detection neck than that provided by SPPF.

**Figure 11:**
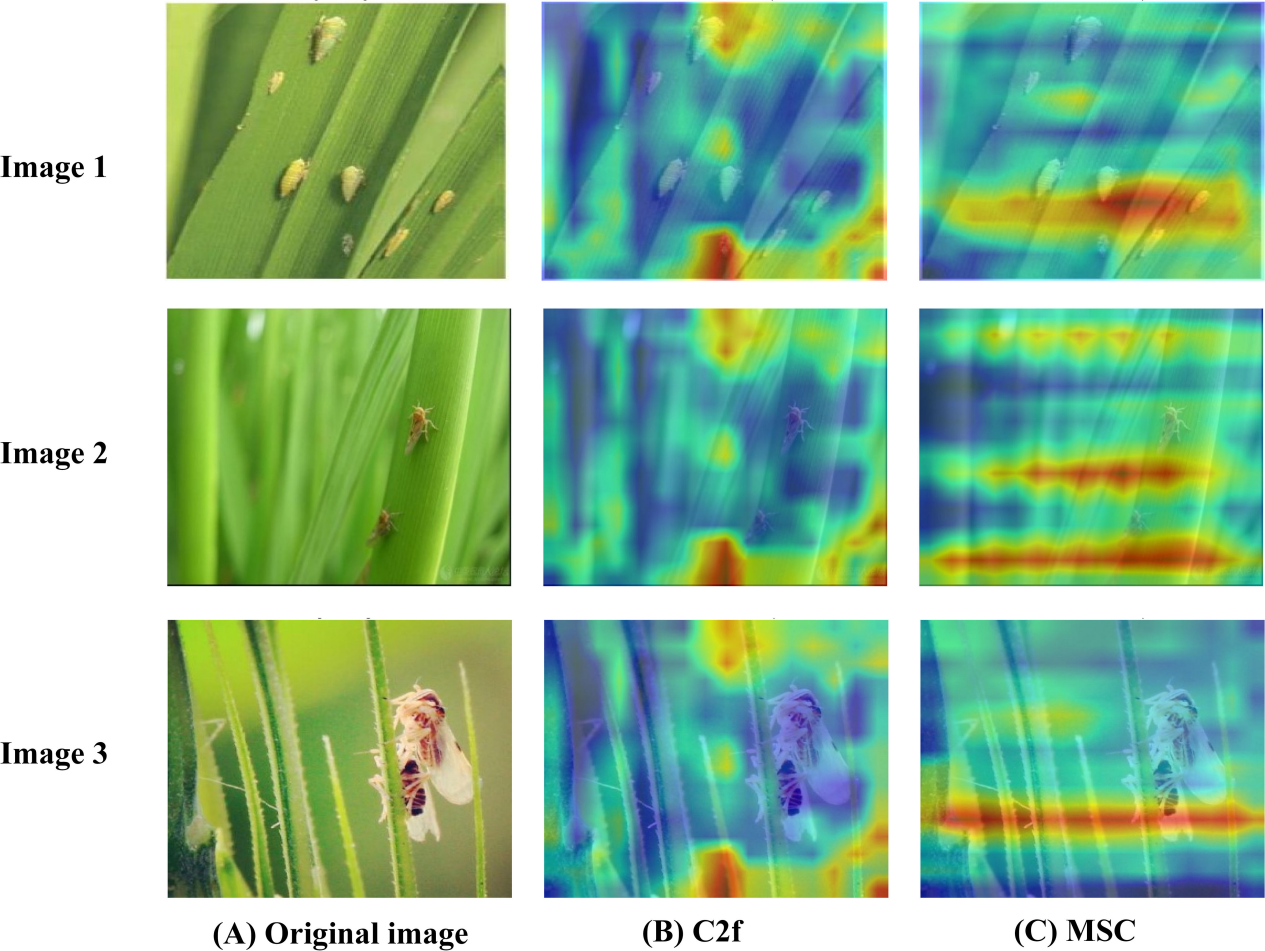
CAM comparison of C2f and MSC. **(A)** Original image. **(B)** C2f heatmap. **(C)** MSC heatmap.

As depicted in **Figure 11**, a further analysis was conducted on the feature heatmaps output at the same network depth by the standard C2f module and our proposed MSC module. When processing images with complex background textures that are similar to the pest targets, the heatmap of the standard C2f module exhibits large-scale redundant attention on background interference. Its activation area is diffuse and fails to precisely focus on the pest itself. Considering the complex background characteristics of rice paddies, where numerous stems and leaves closely resemble pests in both color and shape, insufficient attention to key target regions may introduce significant interference and increase the risk of false detections. In contrast, the proposed MSC module, through its multi-receptive field parallel branches, can simultaneously capture both the fine-grained details and the broader context of the target. As a result, the activation area in its heatmap is more compact and more accurately focused on the pest target, effectively distinguishing between critical and non-critical regions.

**Figure 12:**
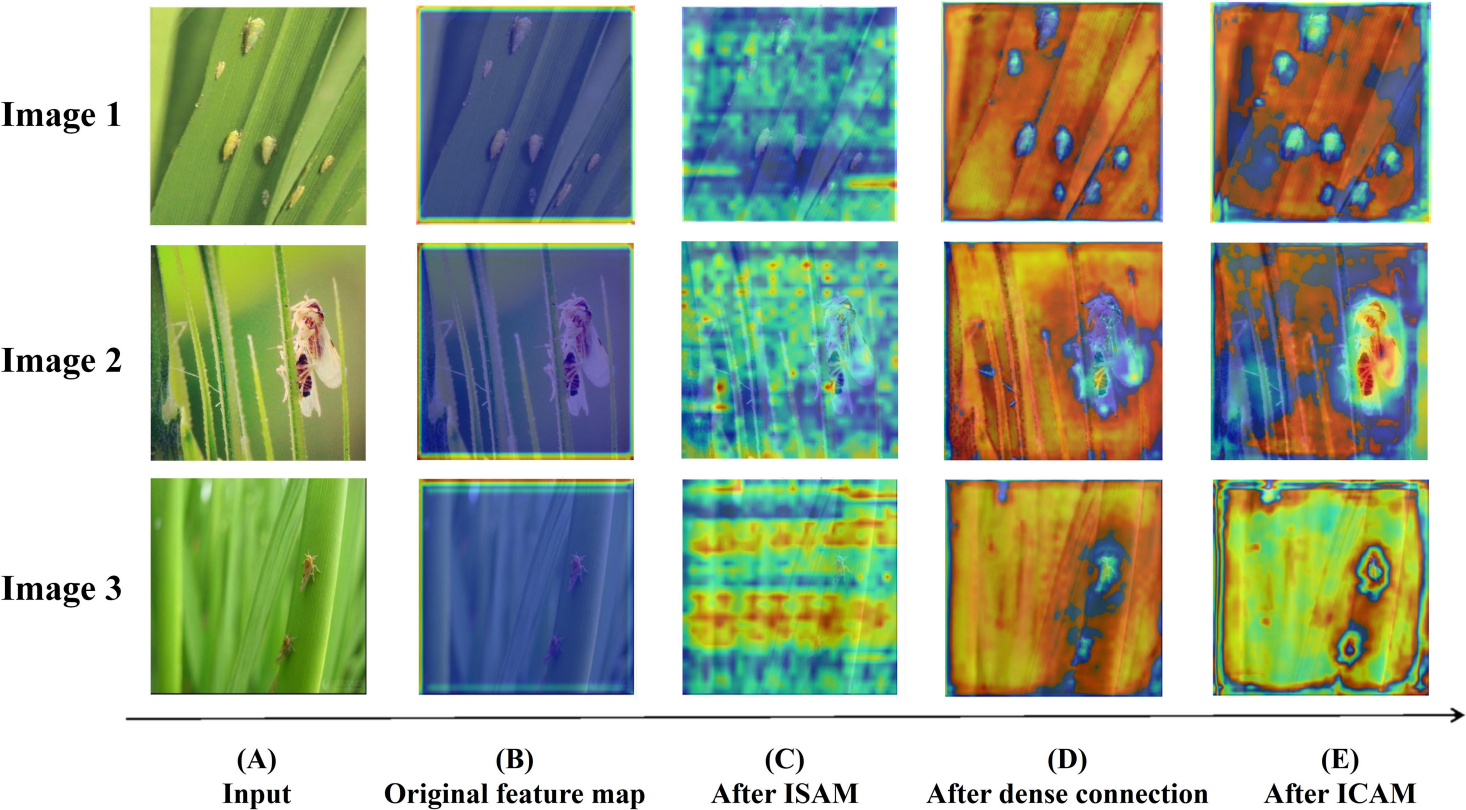
Visualization of the feature map’s evolution at different stages of the HADF-Net.

Finally, **Figure 12** illustrates the complete process of end-to-end optimization and progressive refinement that the HADF-Net performs on the feature maps. The initial feature map generated by standard convolution (**Figure 12B**) exhibits a very weak and diffuse activation response, with the entire heatmap showing large blue areas of low response. The process begins in the backbone with the ISAM. After processing (**Figure 12C**), the activation pattern transforms into a coherent region with a holistic perception of the target, indicating that the model has initially constructed long-range contextual information. Building on this, the enhanced features enter the dense aggregation neck for fusion. At this stage, the feature activation area (**Figure 12D**) becomes more compact and enriched with detailed information from different levels, which visually demonstrates the effectiveness of HADF-Net’s dense topology in counteracting information dilution. As the culmination of the workflow, this information flow is integrated by the ICAM, where attention is ultimately focused on the most discriminative features that the prediction task relies upon. This forms highly refined activation hotspots with clear contours (**Figure 12E**). This series of progressive changes clearly showcases how HADF-Net upgrades feature processing from a passive pipeline into an active, multi-level, synergistic, and intelligent perception system that transitions from global enhancement to precise localization.

## 4 Mobile application for pest recognition

**Figure 13:**
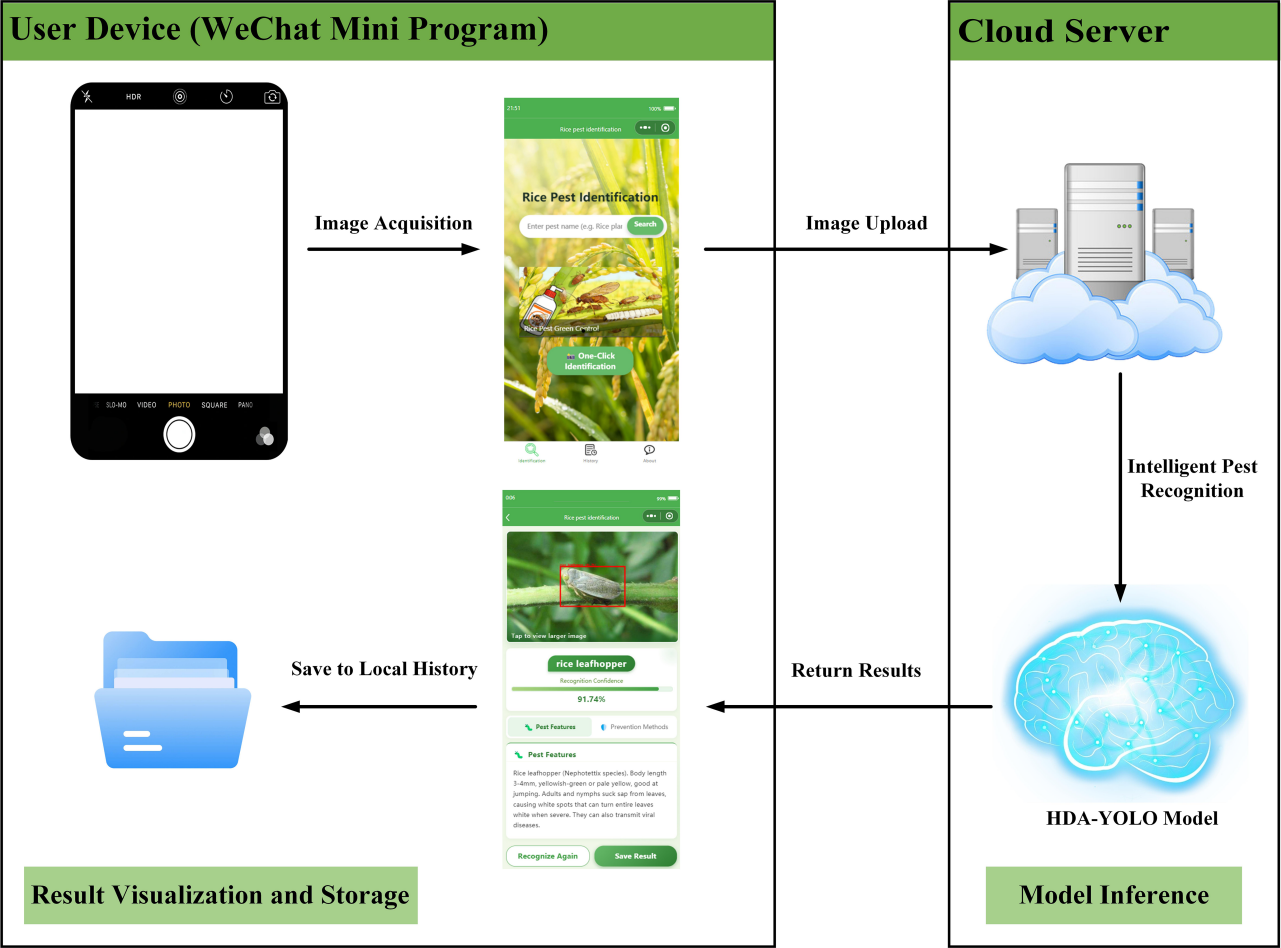
The workflow of the intelligent pest recognition system based on the WeChat Mini Program.

Based on the proposed improved model for rice pests, an agricultural pest recognition application was developed in the form of a WeChat Mini Program. This application integrates the lightweight deep learning model with a user-friendly interface to achieve intelligent recognition and visual analysis of rice pest images. The system follows the detection workflow illustrated in **Figure 13**: First, a farmer captures or uploads an image of a field crop through the Mini Program. Subsequently, the image is transmitted to a cloud server for real-time detection. Finally, the recognition result, along with corresponding pest control recommendations, is returned to the user’s device and automatically saved to the local history records.

The system adopts a multi-terminal collaborative architecture. The WeChat Mini Program frontend is developed based on WXML/WXSS and integrates camera access and image preview functionalities. The backend program is based on the Flask framework, developed using PyCharm, and deployed on a rented Tencent Cloud server, providing RESTful API endpoints. The history module utilizes a local caching strategy, supporting the persistent storage and visual retrieval of detection data. Currently, the system supports the recognition of 12 common rice pest species. In addition, it is equipped with a professional knowledge base that provides information on pest morphological characteristics and control methods. Through a simple image upload operation, users can instantly obtain accurate identification results.

**Figure 14:**
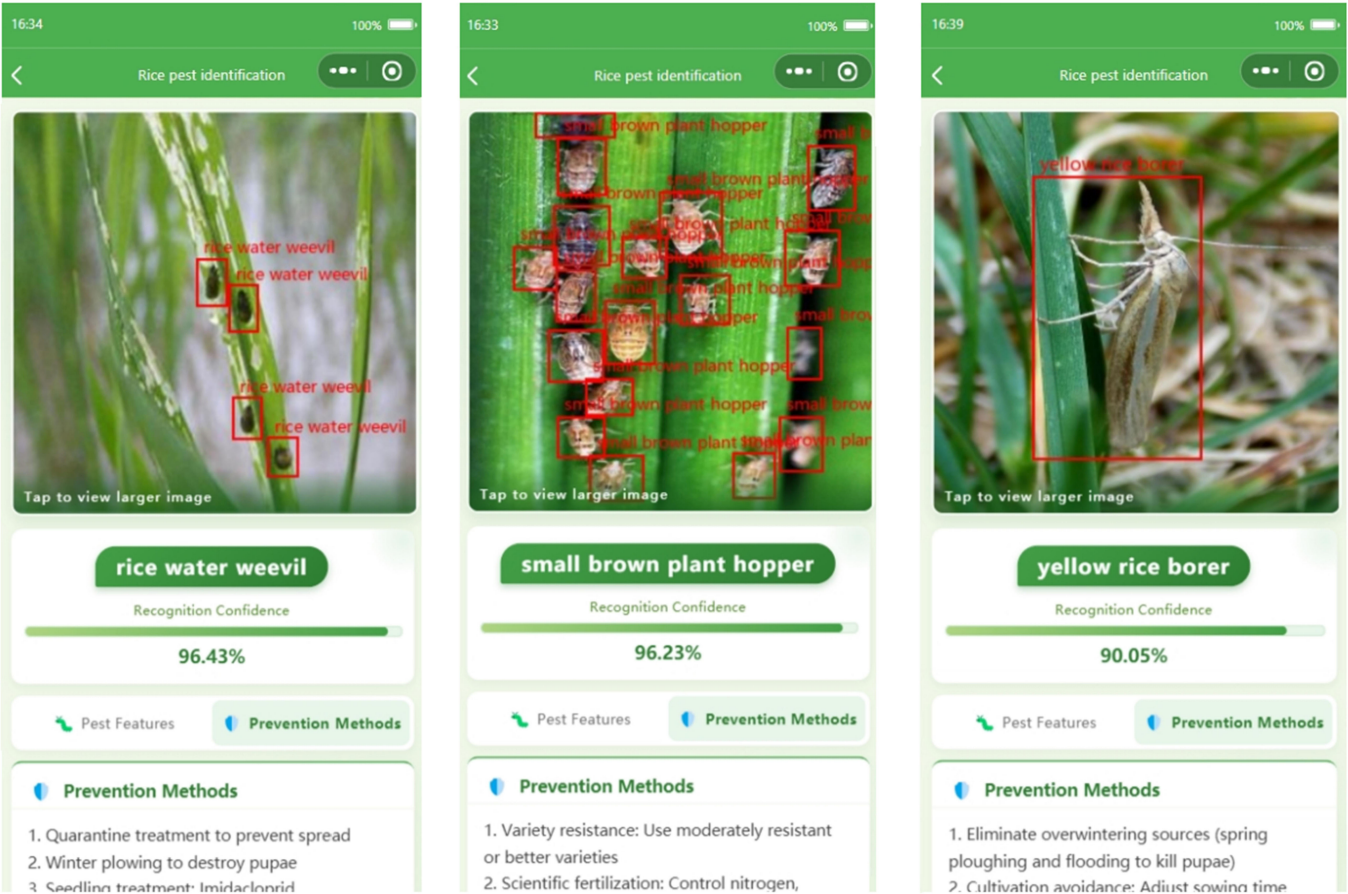
The user interface for displaying detection results in the WeChat Mini Program.

Regarding the display and analysis of detection results, the system interface is shown in **Figure 14**. The results screen intuitively displays the category and bounding box of the target pest in the uploaded image, along with the model’s prediction confidence. It also provides descriptions of the corresponding pest’s morphological characteristics and recommended control measures. Users can click on a result to view detailed information and save it to their history records. Experimental results show that the system achieves an average recognition accuracy of 88.2% on the 12 rice pest classes. For high-frequency pests such as rice planthoppers and rice leaf rollers, the recognition accuracy exceeds 92%, which is sufficient to meet the demands of practical field applications. Future work aims to improve transmission efficiency by implementing image tiling and to cover a broader range of pests, offering farmers more precise field management support.

## 5 Conclusion and future work

Existing lightweight detection models often suffer from insufficient accuracy when detecting multi-scale and easily camouflaged rice pests in complex agricultural scenarios. To address this issue, this study proposes a novel and deeply optimized YOLOv8-based object detection model, termed HDA-YOLO. The core of this model is an end-to-end synergistic optimization framework. It jointly optimizes three key stages: feature extraction, feature fusion, and feature interpretation, thereby enhancing the fidelity and interaction efficiency of information flow across the entire network. The model systematically introduces several synergistic technical innovations. In the backbone network, a high-fidelity feature foundation is constructed using the ADDS module and the MCPF module. These foundational components provide high-quality input to the core HADF-Net, which significantly boosts model performance through dynamic fusion and interpretation. Simultaneously, to further enhance the network’s multi-scale analysis capabilities during feature fusion, the C2f module in the neck was upgraded to a MSC module.

Extensive experimental results have fully validated the effectiveness of the proposed solution. On our self-built rice pest dataset, HDA-YOLO achieved a mAP@50 of 90.0% and an F1-score of 88.2%, representing significant improvements of 2.4 and 3.8 percentage points over the baseline model, respectively, and demonstrated particularly excellent performance in small object detection. Furthermore, the feature map visualization analysis intuitively confirmed the synergistic effect of the various improved modules in enhancing feature discriminability and precisely focusing on targets. Ultimately, the HDA-YOLO model was integrated into a WeChat Mini Program-based automatic rice pest identification system, demonstrating its high accuracy and significant application potential for intelligent agricultural monitoring scenarios.

While HDA-YOLO demonstrates superior performance, this study acknowledges certain limitations. In the pursuit of maximum identification accuracy, the model’s computational load has increased moderately. Currently, experiments are primarily focused on a single-crop dataset, and the model’s robustness across diverse datasets and extreme environmental variations—such as severe occlusion, challenging illumination, and motion blur—requires further reinforcement. Additionally, energy management and long-term stability in resource-constrained scenarios have not yet been comprehensively investigated. Future research will prioritize model quantization, multi-environment data augmentation, and domain adaptation techniques. These efforts aim to continuously enhance the model’s generalization capability and application potential in complex agricultural scenarios, ultimately providing more robust technical support for the advancement of intelligent agriculture.

## Data Availability

The data analyzed in this study is subject to the following licenses/restrictions: The data will be made available upon reasonable request and with permission from the corresponding author. Requests to access these datasets should be directed to Xinhui Zhou, zxhui@henu.edu.cn.
